# Impact of T-RFLP data analysis choices on assessments of microbial community structure and dynamics

**DOI:** 10.1186/s12859-014-0360-8

**Published:** 2014-11-08

**Authors:** Nils Johan Fredriksson, Malte Hermansson, Britt-Marie Wilén

**Affiliations:** Department of Medical Biochemistry and Cell Biology, Institute of Biomedicine, Sahlgrenska Academy, University of Gothenburg, Gothenburg, Sweden; Department of Chemistry and Molecular Biology, University of Gothenburg, Gothenburg, Sweden; Department of Civil and Environmental Engineering, Water Environment Technology, Chalmers University of Technology, Gothenburg, Sweden

**Keywords:** Terminal restriction fragment polymorphism analysis, Alignment, Normalization, Bacterial community structure and dynamics

## Abstract

**Background:**

Terminal restriction fragment length polymorphism (T-RFLP) analysis is a common DNA-fingerprinting technique used for comparisons of complex microbial communities. Although the technique is well established there is no consensus on how to treat T-RFLP data to achieve the highest possible accuracy and reproducibility. This study focused on two critical steps in the T-RFLP data treatment: the alignment of the terminal restriction fragments (T-RFs), which enables comparisons of samples, and the normalization of T-RF profiles, which adjusts for differences in signal strength, total fluorescence, between samples.

**Results:**

Variations in the estimation of T-RF sizes were observed and these variations were found to affect the alignment of the T-RFs. A novel method was developed which improved the alignment by adjusting for systematic shifts in the T-RF size estimations between the T-RF profiles. Differences in total fluorescence were shown to be caused by differences in sample concentration and by the gel loading. Five normalization methods were evaluated and the total fluorescence normalization procedure based on peak height data was found to increase the similarity between replicate profiles the most. A high peak detection threshold, alignment correction, normalization and the use of consensus profiles instead of single profiles increased the similarity of replicate T-RF profiles, i.e. lead to an increased reproducibility. The impact of different treatment methods on the outcome of subsequent analyses of T-RFLP data was evaluated using a dataset from a longitudinal study of the bacterial community in an activated sludge wastewater treatment plant. Whether the alignment was corrected or not and if and how the T-RF profiles were normalized had a substantial impact on ordination analyses, assessments of bacterial dynamics and analyses of correlations with environmental parameters.

**Conclusions:**

A novel method for the evaluation and correction of the alignment of T-RF profiles was shown to reduce the uncertainty and ambiguity in alignments of T-RF profiles. Large differences in the outcome of assessments of bacterial community structure and dynamics were observed between different alignment and normalization methods. The results of this study can therefore be of value when considering what methods to use in the analysis of T-RFLP data.

**Electronic supplementary material:**

The online version of this article (doi:10.1186/s12859-014-0360-8) contains supplementary material, which is available to authorized users.

## Background

Knowledge about microbial communities and the factors governing microbial community composition is fundamental for our understanding of ecology, but also for biotechnological applications such as wastewater treatment [1]. Since isolation and cultivation is usually only successful for a fraction of the bacterial species present in an environmental sample (e.g. [2]), DNA-based methods are routinely used to describe microbial communities. DNA fingerprinting, gene sequencing, and in recent years, next-generation sequencing technologies enables descriptions of microbial communities at different resolution, where the latter provides an unprecedented level of detail. Although fingerprinting methods, such as terminal restriction fragment polymorphism (T-RFLP) (e.g. [3]), are trumped by next-generation sequencing technologies when it comes to describing the depth of microbial communities, numerous studies comparing the two methods have shown that the same conclusions can be drawn from both approaches, both with regard to community structure [4-7] and dynamics [8-10] of the community. It can also be argued that the advantage of traditional fingerprinting techniques is the ability to analyze a high number of samples at a low cost [11], thus ensuring proper replication and statistical power. In conclusion, despite the continuously decreasing cost and the popularity of next-generation sequencing, fingerprinting techniques such as T-RFLP are still relevant and an important tool for studies of microbial communities.

In a T-RFLP analysis, the gene of interest, typically the 16S rRNA gene, is amplified by PCR where one or both of the primers are labeled with a fluorescent marker. The gene is then digested by a restriction enzyme and the restriction fragments are separated by polyacrylamide or capillary gel electrophoresis. The terminal restriction fragments (T-RFs) are detected by an automated DNA sequencer and the lengths of the T-RFs are estimated. The resulting T-RFLP fingerprint of a community is a set of T-RFs, referred to as a T-RF profile. The length of a T-RF depends on the position of the restriction enzyme recognition sites and different T-RF lengths therefore represent different gene sequences.

Differences in microbial community composition between samples are assessed by comparing the presence and relative abundances of T-RFs in the T-RF profiles. To be able to accurately interpret differences in T-RF profiles as differences in community composition it is important to know the variability of the method and how effective different analysis methods are in reducing variations and maintaining reproducibility. An important part of the analysis is to distinguish true T-RFs from false T-RFs derived from noise peaks or artifacts. One way to do this is by the application of a peak detection threshold (PDT), either when the peaks are detected and T-RF sizes are estimated, or afterwards, on the already generated data. In the literature, the range of applied PDTs varies from low (25) [12], accepting most detected T-RFs, to high (200) [13], only considering peaks with a high fluorescence intensity. This range may be due to differences in the obtained fluorescence intensity of the peaks and background noise levels between different analytical platforms. In this study T-RFLP analyses were carried out using the 3730 DNA Analyzer (Applied Biosystems) with a typical baseline noise level between 20 and 30 fluorescence units. Here we evaluate the effect of applying an intermediate (50) or high (100) PDT on the characteristics of the T-RF profiles and on the subsequent comparisons of T-RF profiles. Other important steps in the analysis of T-RFLP data which affects the reproducibility is the alignment of T-RFs and the normalization of T-RF profiles, both of which we evaluate here.

Alignment of T-RFs is the process by which it is determined which T-RFs that are the same in two or more samples. This may seem straightforward but due to the large variation in the estimated sizes of the T-RFs it can be both time-consuming and difficult to do in an accurate way, in particular when analyzing large datasets with high number of T-RFs. Various approaches to the alignment of T-RFs have been presented in the literature (e.g. [12,14,15]) and tools for automatic alignment has been made available (e.g. [14-16]). However, although these methods are sound and can be rationally argued for, in our experience there are still problems with the alignment that remain unsolved, mainly due to the variation in estimated T-RF sizes. In this study we present a novel approach to evaluate and correct the alignment, which adjusts for differences in the estimated sizes of the T-RFs.

Standardization, or normalization, is important to remove differences between T-RF profiles due to differences in the amount of DNA that has been loaded on the gel. As for T-RF alignment, several methods for normalization have been presented (e.g. [12,15,17-19]). The normalization procedures, as well as the subsequent comparison of T-RF profiles, can be based on either peak height or peak area data. The use of peak areas can be motivated by the observation that peak width increases and peak height decreases with increasing migration time through the gel [20]. Because of this, long fragments will result in broad and low peaks and will be underestimated if the relative abundances of T-RFs are based on peak heights. However, if peak areas are used, alterations in the relative abundances of the T-RFs may be produced by overlapping peaks. An evaluation also showed that the use of peak heights more accurately described the relative abundances of T-RFs derived from samples with defined amounts of different templates [21]. To evaluate how the definition of total fluorescence affects the outcome, in this study we use both peak height and peak area, both in the comparisons of different normalization procedures and in the comparisons of T-RF profiles.

Some normalization methods have previously been evaluated. Osborne *et al.* [19] compared three different normalization methods: the constant percentage threshold procedure [18], the total fluorescence normalization procedure [12] and the variable percentage threshold procedure [19]. However, all three methods were based on peak area data and only three pairs of replicate samples were used to evaluate how well the normalization methods performed. Moreover, it was not evaluated for how large variations in the amount of loaded DNA the normalization methods were effective. In this study we evaluate two different normalization procedures (the total fluorescence normalization procedure [12] and the fixed percentage threshold procedure [22]) and variants thereof and assess how large differences in initial total fluorescence that can be adjusted for. Comparisons are also made with a third method, the noise filtering method by Abdo *et al.* [15].

The aims of this study are to improve available automatic alignment procedures, to evaluate the efficiency of different normalization methods and to evaluate the effect of combinations of PDT and alignment and normalization strategies on reproducibility. Furthermore, the impact of the alignment and normalization methods on the results of comparative analyses of T-RF profiles is also evaluated. Do the method choices make a great difference in the general interpretations of the results, or do the methods only change the results at a finer, perhaps negligible, level?

The evaluations are done using four different datasets. A dilution series with DNA concentrations from 17% to 100% is used to investigate the relation between total fluorescence and sample concentration, the effect of using single or consensus profiles and to evaluate the range of efficiency of a normalization procedure. A set of 51 samples loaded twice is used to evaluate the variations in T-RF size estimations and the efficiency of different normalization methods. A set of T-RF profiles derived from DNA-extraction replicates and PCR replicates is used to evaluate the efficiency of different combinations of peak detection threshold, alignment correction, and normalization methods. Finally, a dataset from a longitudinal study of the bacterial community in an activated sludge wastewater treatment plant (WWTP) is used to evaluate the impact of different treatment combinations on subsequent comparative analyses of the T-RF profiles.

## Results and discussion

### Estimation of T-RF sizes

The estimation of T-RF sizes based on the migration time through the gel depends on the length, the nucleotide composition and the secondary structure of the T-RFs [23,24]. Estimated T-RF lengths have been reported to be between one and eight bases longer or shorter than the true lengths [23,24]. Here we show that there is also a run-to-run variation in the estimation of the T-RF sizes. A set of 51 samples was loaded twice on the capillary gel and the resulting T-RF profiles from the two loadings were compared. The differences in the estimated T-RF sizes between loading duplicates varied between 0 and 0.97 bases. The same variation range was observed for T-RFs of all sizes. The average difference was 0.21 ± 0.19 bases and for 90% of the T-RFs the size difference between the runs was lower than 0.5. Thus, in most cases, the size variation is very low and will not affect the alignment and subsequent analyses. However, in the cases where the size difference is above 0.5, the alignment may be affected.

### Alignment of T-RFs

Before a comparison of two T-RF profiles can be made they need to be aligned, by comparing the sizes of the T-RFs present in the profiles. This process of comparing T-RFs is often referred to as T-RF binning, by placing T-RFs of similar sizes in alignment bins. The easiest way to bin the T-RFs would perhaps be to convert the T-RF sizes, which are given with various decimals, to integers and then place all T-RFs of the same size in the same bins. However, two T-RFs of size 134.4 and 134.6 bases would then be added to different bins instead of the same. To enable a more accurate and faster alignment more complex automatic binning procedures are used where T-RFs are binned together if the distance between them is smaller than a pre-defined value (e.g. [14,15]).

In this study, the moving average procedure described by Smith *et al.* [14] was used. For a small number of samples the automatic binning generally works well. However, for a large number of samples, with a high number of T-RFs that are close in size, binning can be problematic due to the variation in size estimations and the resulting alignment needs to be checked before further analysis. In the T-RFLP data analysis work flow used in this study the alignment is evaluated by classifying the alignment bins as ambiguous or correct. Alignment bins are classified as ambiguous if any of the T-RFs in the bin are within the given alignment range (for example 1 base) of a T-RF in another alignment bin. For alignments of only two profiles, as in the comparison of loading duplicates above, ambiguous alignment bins can be easily corrected, by binning the T-RFs that are most similar in size (Additional file 1: Table S1). For the alignment of more than two profiles the same approach does not work (Additional file 1: Table S2).

**Figure 1:**
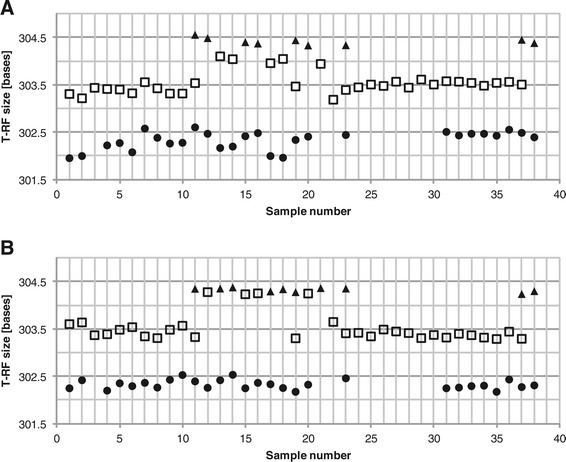
**Example of ambiguous alignment bins and correction using the systematic shift procedure.** The different symbols represent the alignment bins generated by the automatic binning procedure. **Panel A** shows the original alignment and **Panel B** the alignment after correction for systematic shifts in the estimation of T-RF sizes.

For replicate profiles, as in the examples of Additional file 1: Tables S1 and S2, ambiguous alignment bins can be easily corrected, since ideally, the same T-RFs should be present in all profiles. However, when the T-RF profiles are derived from different samples and the purpose of the analysis is to detect and quantify similarities or differences in community composition, the alignment of the T-RFs is harder to correct. To illustrate the alignment problems that can arise, 38 T-RF profiles generated from activated sludge samples were analyzed and automatically aligned using the moving average procedure as described by Smith *et al* [14]. Due to the large variation in size estimation and the high number of T-RFs with little size difference, 40 of 59 alignment bins were classified as ambiguous. Figure 1 (panel A) shows an example of alignment bins classified as ambiguous.

### Systematic shift correction

**Figure 2:**
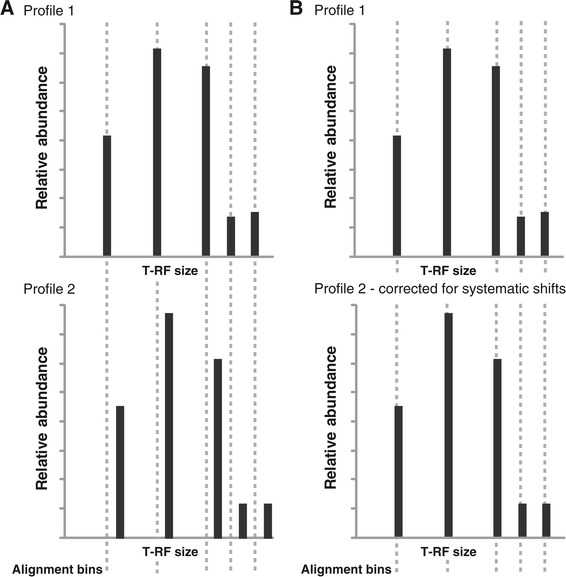
**Concept of systematic shift correction.** All T-RFs in profile 2 are estimated as a little longer than the T-RFs in profile 1 **(Panel A)**. A correction for the systematic shift facilitates the alignment **(Panel B)**.

The differences in the size estimations of the T-RFs are often systematic, i.e. all T-RFs in one profile are estimated as a little longer than the T-RFs in another profile. For example, for the 38 activated sludge samples there are 703 possible pair-wise comparisons of T-RF profiles. Of these, 373 comparisons, slightly more than half, showed a systematic shift in the size estimations. By adjusting the T-RF sizes and correcting for the systematic shift the alignment can be improved. Figure 2 illustrates the concept of systematic shift correction.

In Figure 1 (panel B) it can be seen how the adjustment for systematic shifts reduces the variation in T-RF sizes between samples and allows for a better binning. Before the adjustment it is uncertain if there should be one or two alignment bins, and if two, where the T-RFs should be binned. The systematic shift correction removes this uncertainty. Note that, as stated above, there is not a systematic shift in the size estimations for all pair-wise comparisons so the systematic shift correction is not valid for all T-RF profiles. However, in the given example in Figure 1, the profiles with the shortest T-RFs all display a systematic shift towards the profiles with the longest T-RFs. It should also be noted that even though corrections for systematic shifts do help in resolving many ambiguous alignment bins, it does not always succeed.

In the time series dataset there were 59 alignment bins in the original alignment. 33 of these, more than half, were determined to be ambiguous after initial inspection. By using the systematic shift correction as an aid to objectively resolve the alignment, the total number of bins was reduced to 54, with only 17 remaining ambiguous.

### Total fluorescence and number of observed T-RFs

**Figure 3:**
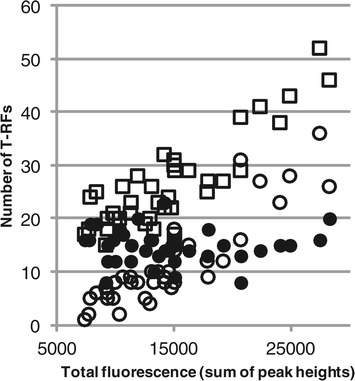
**Number of T-RFs versus total fluorescence.** The total number of T-RFs (squares), the number of T-RFs with a relative abundance above 1% (Filled circles) and below 1% (empty circles) are plotted against the total fluorescence of 38 T-RF profiles from activated sludge samples. Total fluorescence was defined as the sum of all peak heights in a profile and the relative abundance of a T-RF was calculated as the peak height of the T-RF divided by the total fluorescence.

There was a large variation in total fluorescence and in the number of observed T-RFs between the 38 activated sludge samples in the time series dataset. The total number of T-RFs was clearly related to the total fluorescence: a high total fluorescence corresponds to a high number of T-RFs (Figure 3). However, division of the T-RFs into two groups, T-RFs with a relative abundance above and below 1%, showed that a high total fluorescence only increase the number of T-RFs of low relative abundance. The same pattern was observed independent if the total fluorescence was defined as the sum of peak heights or peak areas. The relation between total fluorescence and number of T-RFs has been observed previously by for example Sait *et al.* [18]. However, in their data the threshold where an increase in total fluorescence no longer correlated with an increased number of T-RFs was 5%, and not 1% as for the data presented here.

**Figure 4:**
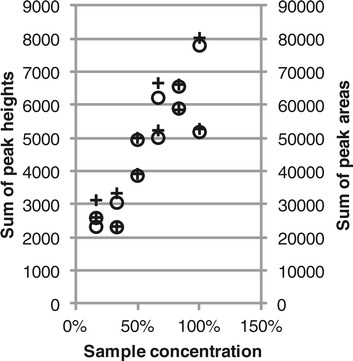
**Total fluorescence versus DNA concentration.** Total fluorescence was calculated as the sum of peak heights (circles) or peak areas (crosses).

The analysis of a series of dilutions of the same sample showed that the total fluorescence was related to the DNA concentration in the sample (Figure 4). Osborn *et al.* [25] also investigated the relation between total fluorescence and sample DNA concentration with the same results. The experiment was repeated here to provide data that could be used to evaluate how the differences in total fluorescence affect comparisons of the T-RF profiles and the efficiency of the normalization methods.

Analysis of the loading duplicates dataset showed that differences in total fluorescence is not only dependent on concentration differences but also on the loading itself. The average difference in total fluorescence (sum of peak heights) between two duplicates, as a percentage of the highest total fluorescence, was 17 ± 7% and the maximum difference was 33%.

**Figure 5:**
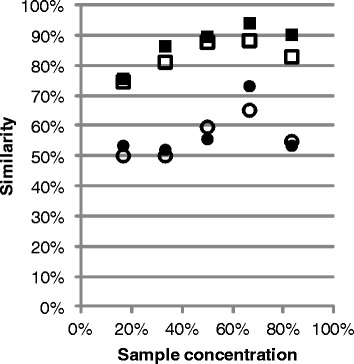
**Similarity versus DNA concentration.** Jaccard (circles) and Bray-Curtis (squares) similarities between the profiles of all diluted samples in the dilution series and the profile of the non-diluted sample. Each sample was loaded twice on the capillary gel. Filled symbols represent the first loading and empty symbols the second loading. The profiles were analyzed using a PDT of 50.

As the number of T-RFs in a profile depends on the total fluorescence of the profile, differences in total fluorescence can affect comparisons of T-RF profiles. The profiles in the dilution series were compared with the profiles of the undiluted sample using Jaccard and Bray-Curtis similarities. For concentrations between 17% and 67% the similarity increased with increasing concentration (Figure 5). However, the profiles of the sample with concentration 83% showed lower similarities than the profiles of the 50% sample, due to the absence of several low abundance T-RFs.

**Figure 6:**
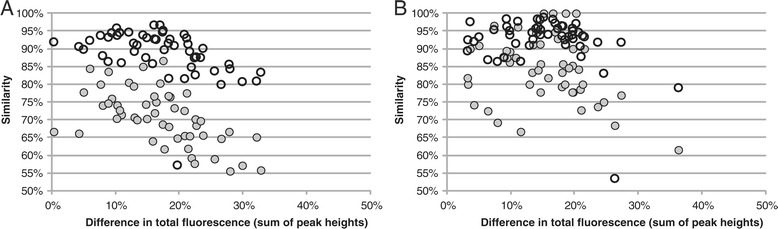
**Similarity versus difference in total fluorescence.** Bray-Curtis (empty symbols) and Jaccard (filled symbols) similarities of 51 loading duplicates plotted against difference in total fluorescence (relative the highest total fluorescence of the two duplicates) based on peak heights. The T-RF profiles were analyzed with a PDT of 50 **(Panel A)** and a PDT of 100 **(Panel B)**.

In the loading duplicates dataset, the similarities decreased with increasing differences in total fluorescence when a PDT of 50 was applied (Figure 6). The relatively low similarities were due to a high number of T-RFs not observed in both loadings. Generally, both profiles had T-RFs not present in the other, not only the profile with the highest total fluorescence. Most T-RFs that were only observed in one duplicate were of low relative abundance, on average 0.9% ± 1.8%. As a result, the Bray-Curtis similarities, which are calculated using the relative abundances of the T-RFs, were higher than the Jaccard similarities, which put equal weight on all T-RFs (Figure 6). The same pattern was seen when the analysis was based on peak heights as when it was based on peak areas.

Applying a higher PDT removes the T-RFs with a low relative abundance. With a PDT of 100, the average proportion of T-RFs only observed in one of the loading duplicates was reduced from 29 ± 8% to 16 ± 9% of all T-RFs in a loading duplicate pair. The similarities were also increased for 47 of the 51 profile pairs and the decrease in similarities for greater differences in total fluorescence was less marked (Figure 6). The same results were obtained independent if the analysis was based on peak heights or peak areas. Blackwood *et al.* [26] investigated the effect of different PDTs on subsequent ordination analyses. However, contrary to the results presented here, they found that increasing the PDT from 50 to 100 or 200 decreased the similarity between replicates.

It is noteworthy that in the analysis using a PDT of 50, one unique T-RF had a relative abundance of 43%. However, that T-RF was not observed in any of the loadings of two other PCR replicates of the same sample, and was therefore most likely an artifact.

### Normalization

Five normalization procedures, three based on the total fluorescence normalization procedure [12] (TFN-heights, TFN-areas and TFN-areas-LT) and two using a fixed percentage threshold procedure [22] (FPT-heights and FPT-areas), were evaluated using the loading duplicates dataset. The profiles were normalized pairwise and the profile pairs were then compared. The normalization procedures differed in the number of T-RFs they removed and in the resulting Jaccard and Bray-Curtis similarities between the duplicates (Table 1). Before treatment the number of T-RFs increased with increasing total fluorescence. This pattern was still seen after treatment with TFN-areas and TFN-areas-LT, but not with TFN-heights, FPT-heights or FPT-areas (Additional file 2: Figure S1). The TFN-areas procedures removed hardly any T-RFs and did not improve the Jaccard similarities. However, the Bray-Curtis similarities were significantly increased. The TFN-heights treatment reduced the number of T-RFs the most but also increased both the Jaccard and Bray-Curtis similarities the most. Both of the FPT treatments reduced the number of T-RFs but for FPT-areas there was no significant difference in similarities compared to the untreated dataset. FPT-heights only improved the Bray-Curtis similarities. Although removing more than half of the T-RFs, the TFN-heights treatment appear to be the best as it was the only treatment that increased the similarity between the duplicates, both when equal weight was given to all T-RFs (Jaccard similarities) and when relative abundances were taken into account (Bray-Curtis similarities).

**Table 1:** **Number of T-RFs and Jaccard and Bray-Curtis similarities of the loading duplicates dataset after a PDT of 50 and normalization**

**Treatment**	**NT-heights**	**NT-areas**	**TFN-heights**	**TFN-areas**	**TFN-areas-LT**	**FPT 1% -heights**	**FPT 1% -areas**
**No of T-RFs**	43 ± 15	43 ± 15	18 ± 8*	42 ± 15	42 ± 15	21 ± 7*	22 ± 7*
**Jaccard**	71 ± 8%	71 ± 8%	84 ± 11%*	71 ± 8%	71 ± 8%	78 ± 14%*	74 ± 13%
**Bray-Curtis**	89 ± 6%	87 ± 6%	96 ± 4%**	93 ± 3%*	87 ± 6%	91 ± 8%	88 ± 7%

All values are average values ± the standard deviation. The number of T-RFs is the average for all 102 profiles. The Jaccard and Bray-Curtis similarities are the averages similarities of all 51 duplicate pairs. The T-RF profiles were analyzed with a PDT of 50. Descriptions of the treatments can be found in the Methods section. *denotes a significant difference from NT-heights. **denotes a significant difference from all other treatments.

**Figure 7:**
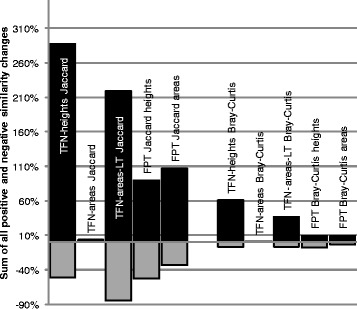
**Change in similarity of loading duplicates after normalization.** The loading duplicate dataset was normalized using five different procedures (described in the Methods section). The Jaccard and Bray-Curtis similarities after normalization were compared to the similarities before normalization. Sum of all positive (black columns) and negative (gray columns) changes in similarity after normalization are shown. The T-RF profiles were analyzed with a PDT of 100.

With an applied PDT of 100 there was no significant difference between normalization treatments for number of T-RFs, Jaccard or Bray-Curtis similarity (Table 2), as many of the T-RFs not present in both profiles had already been removed. However, although the similarities of the entire dataset after treatment were not significantly different from the untreated dataset, some of the procedures did increase the similarity for many of the profiles (Table 3). The TFN-heights procedure increased the similarities more than the other treatments (Figure 7).

**Table 2:** **Number of T-RFs and Jaccard and Bray-Curtis similarities of the loading duplicates dataset after a PDT of 100 and normalization**

**Treatment**	**NT-heights**	**NT-areas**	**TFN-heights**	**TFN-areas**	**TFN-areas-LT**	**FPT 1% -heights**	**FPT 1% -areas**
**No of T-RFs**	17 ± 9	17 ± 9	16 ± 9*	17 ± 9	16 ± 9	14 ± 6	14 ± 6
**Jaccard**	84 ± 9%	84 ± 9%	89 ± 11%*	84 ± 9%	87 ± 11%	85 ± 10%*	86 ± 10%
**Bray-Curtis**	93 ± 7%	92 ± 6%	94 ± 7%*	92 ± 6%*	93 ± 6%	93 ± 7%*	92 ± 6%

All values are average values ± the standard deviation. The number of T-RFs is the average for all 102 profiles. The Jaccard and Bray-Curtis similarities are the averages similarities of all 51 duplicate pairs. The T-RF profiles were analyzed with a PDT of 100. Descriptions of the treatments can be found in the Methods section. There was no significant difference between the treatments. All numbers except marked with *were significantly different from the same treatment in the PDT 50 analysis.

**Table 3:** **Count of number of duplicate pairs with increased or decreased similarity after normalization**

**Treatment**	**TFN-heights**	**TFN-areas**	**TFN-areas-LT**	**FPT-heights**	**FPT-areas**
**Increased similarity (Jaccard/Bray-Curtis)**	29/30	1/1	25/26	12/13	19/20
**Decreased similarity (Jaccard/Bray-Curtis)**	8/7	0/0	10/9	9/8	8/7

The T-RF profiles were analyzed with a PDT of 100. The treatments are described in the Methods section.

With a PDT of 100, T-RFs of low relative abundance are removed from the analysis from the start. These T-RFs may well be true T-RFs and could, although of low relative abundance, be of importance in the detection of differences between samples. However, with a PDT of 50 there is a higher risk of including false T-RFs and artifacts. An example is the occasional observation of false T-RFs produced by so called spectral pull-up. Although the fragments of the size standard and the sample are labeled with different fluorophores, a false signal can be detected in the frequency of the sample fluorophore when there is a very strong signal from the fragments of the size standard. However, the false T-RFs produced by spectral pull-up are of low peak heights and are removed when a PDT of 100 is applied (Additional file 2: Figure S2). The PDTs used in the literature varies from low (e.g. 25 [12]) to high (e.g. 100 [25]), presumably depending on the wish to either preserve as many of the true T-RFs as possible or to ensure removal of false T-RFs produced by noise peaks.

Based on the evaluation of the normalization treatments using the loading duplicates dataset, PDT 100 and the TFN-heights normalization procedure seem to be the best analysis combination. The TFN-heights normalization seem to be the best procedure because it made a higher number of duplicate profiles more similar compared to the other treatments (Table 3, Figure 7). A PDT of 100 seems better than a PDT of 50 because it reduces the risk of including false T-RFs in the analysis. In addition, the final number of T-RFs after normalization was not significantly different between the analyses using a PDT of 50 or 100 (Table 4). In other words, although applying a high PDT initially results in a loss of data, after normalization the final number of T-RFs may be the same as if a lower PDT had been applied.

**Table 4:** **Example of remaining number of T-RFs and total fluorescence after treatment**

	**Average no of T-RFs**	**Average total fluorescence**
After PDT 50*	26 ± 9	14693 ± 5486
After PDT 100*	16 ± 5	11018 ± 4517
After normalization**	11 ± 2	5339 ± 18
After alignment***	8 ± 2	3828 ± 438

Average values ± the standard deviation of the number of T-RFs and total fluorescence (sum of peak heights) of the T-RF profiles of 38 activated sludge samples after data treatment. *Replicate profiles were normalized and consensus profiles were generated from two replicate profiles only considering T-RFs present in both. **After normalization of consensus profiles. Both replicate and consensus profiles were normalized using the total fluorescence normalization procedure based on peak heights. ***All T-RFs that could not be unambiguously aligned were removed.

Although applying a high PDT and normalization increased the similarities, it should be noted that the resulting Jaccard and Bray-Curtis similarity after treatment is in most cases lower than 100%. This means that the treatment cannot correct completely for the differences observed in repeated loadings of the same sample.

**Figure 8:**
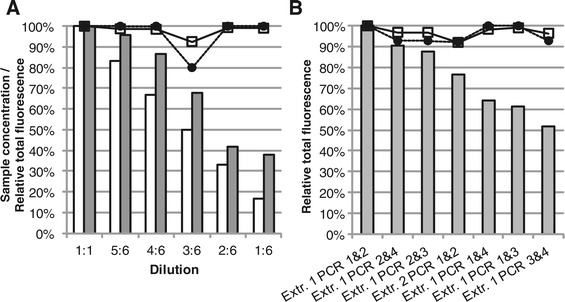
**Similarity versus difference in total fluorescence. Panel A**: Analysis of the dilution series dataset. White columns – sample concentration. Gray Columns – average total fluorescence (sum of peak heights) of loading duplicate profiles before treatment as percentage of the average total fluorescence of the undiluted sample loading duplicates. Circles – Jaccard similarity with dilution 1:1. Squares – Bray-Curtis similarity with dilution 1:1. **Panel B**: Analysis of the DNA-extraction and PCR replicates dataset. Columns – total fluorescence (sum of peak heights) of consensus profiles before normalization as percentage of the highest total fluorescence in the dataset. Circles – Jaccard similarity with sample “Extr. 1 PCR 1&2”. Squares – Bray-Curtis similarity with sample “Extr. 1 PCR 1&2”.

To evaluate how sensitive the normalization procedure is to increasing differences in total fluorescence between samples, the dilution series was analyzed, using the two loading duplicates for each dilution to create consensus profiles. A PDT of 100 was applied and both replicates and consensus profiles were normalized using the TFN-heights procedure. No corrections of the alignments were necessary. The differences in total fluorescence ranged from 4% to 62% (before treatment, total fluorescence as sum of peak heights, difference as percentage of the undiluted sample). Very high similarities were observed between the undiluted sample and all other concentrations (Figure 8). The Bray-Curtis similarities with the non-diluted sample were for all but one dilution higher than 98% and the Jaccard similarities were all but one 100%. The profile with lower similarities, dilution 3:6, had a T-RF not present in any other profile.

In the DNA-extraction and PCR replicate dataset the differences in total fluorescence ranged from 10% to 48% (before treatment, total fluorescence as sum of peak heights, difference as percentage of the profile with the highest total fluorescence). A PDT of 100 was applied and both replicates and consensus profiles were normalized using the TFN-heights procedure. The alignment of the consensus profiles was corrected using the systematic shift correction procedure. After treatment the resulting Jaccard and Bray-Curtis similarities with the profile with the highest total fluorescence were 92% as the lowest. The similarities did not decrease with increasing difference in total fluorescence (Figure 8). The differences between the profiles were due to the presence or absence of two T-RFs of low relative abundance and due to differences in the relative abundance of T-RFs present in all profiles (Figure 8).

In the loading duplicate dataset the differences in total fluorescence ranged from 3% to 36% (before treatment, total fluorescence as sum of peak heights, difference as percentage of the profile with the highest total fluorescence). A PDT of 100 was applied and the duplicates were normalized using the TFN-heights procedure. The resulting Jaccard similarities were between 60% and 100% and Bray-Curtis similarities ranged from 53% to 99%. For profile pairs with higher differences in total fluorescence there were greater increases in similarity after treatment compared to the similarity between non-normalized profiles than for profile pairs with little difference in total fluorescence. However, there was no apparent relation between the final similarity and the initial difference in total fluorescence (Additional file 2: Figure S3).

The efficiency of the normalization procedure does not seem to be directly affected by how large the difference in total fluorescence is, at least not up to 62% differences. However, the similarities are not always increased up to 100%, which should be taken into account in comparisons of community composition.

### Consensus profiles

The observation of differences between loading duplicates before and after normalization, whether artifacts or due to differences in the amount of loaded DNA, highlights the need to use consensus profiles of either loading, enzyme digestion or PCR duplicates instead of relying on single profiles for comparisons of different samples. As suggested by Dunbar *et al.* [12] consensus profiles can be created from replicate profiles by only including T-RFs that are observed in all replicates, and using the average values for sizes, peak heights and peak areas.

The dilution series dataset was used to evaluate the difference between comparisons of samples using single profiles or consensus profiles of loading duplicates (Additional file 1: Table S3). When consensus profiles were used both Jaccard and Bray-Curtis similarities were slightly higher than then when single profiles were used.

### Data processing strategies

To further evaluate the effect of combinations of PDT, alignment correction and different normalization procedures on the comparisons of T-RF profiles, a dataset was created from two DNA-extraction replicate samples, resulting in seven T-RF profile pairs. The dataset was analyzed in 20 different ways, including 3 using the normalization and alignment schemes provided in the *T-REX* software [16] and 1 using the normalization procedure by Abdo *et al.* [15] (included in *T-REX*) combined with the alignment procedure presented in this study. The seven consensus profiles were then compared using Jaccard and Bray-Curtis similarities (Additional file 1: Table S4). As a comparison, the six T-RF profiles that were used to create the dataset were also analyzed as single profiles, without generating consensus profiles.

The highest similarities were obtained by applying a high peak detection threshold, correcting the alignment and normalizing both duplicate profiles and all consensus profiles using the TFN-heights procedure (Additional file 1: Table S4). Note that the lowest similarities using this treatment was 92%, meaning that as in the analysis of loading duplicates, the treatment cannot always correct for all differences introduced by the sample processing.

Both the normalization strategies and the T-RF alignment procedure presented here can be said to be conservative in the sense that only data with a high level of certainty is used. In the normalization procedure, T-RFs are removed from the profiles with a high total fluorescence to avoid differences between profiles caused by differences in the amount of loaded DNA. In the alignment procedure presented here alignment bins that remain classified as ambiguous even after correction for systematic shifts are removed from any further analysis. The reason for doing this is to avoid introducing similarities or differences between T-RF profiles based on erroneous alignment binning. The result is that a lot of information in the original data is sacrificed in order to retain a high reliability and accuracy in the comparisons of the T-RF profiles. Table 4 shows an example of how many T-RFs and how much of the total fluorescence that are lost in the different data treatment steps.

### Community dynamics analysis

**Figure 9:**
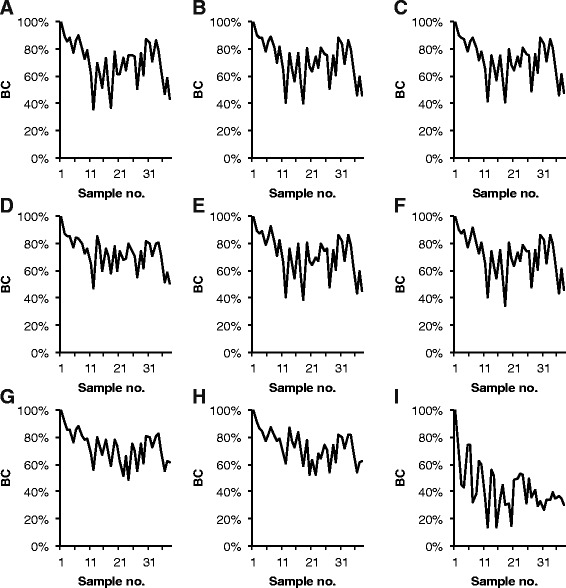
**Community stability: Bray-Curtis similarity between all profiles and the profile of the first sample in the time series.** The data was treated in nine different ways before calculation of Bray-Curtis similarities (BC). **Panel A**: PDT50 TFN-A, **B**: PDT50 TFN-H, **C**: PDT 50 NoNorm, **D**: PDT50 NoNorm, NoAlCorr, **E**: PDT100 TFN-H, **F**: PDT100 TFN-H RepNorm, **G**: TRex-A, **H**: TRex-H, **I**: TRex-H Round-up. The treatments are described in Additional file 1: Table S5.

**Figure 10:**
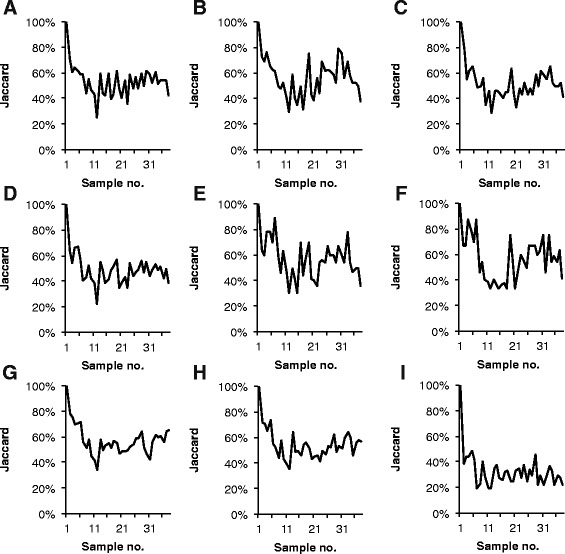
**Community stability: Jaccard similarity between all profiles and the profile of the first sample in the time series.** The data was treated in nine different ways before calculation of Jaccard similarities (Jaccard). **Panel A**: PDT50 TFN-A, **B**: PDT50 TFN-H, **C**: PDT 50 NoNorm, **D**: PDT50 NoNorm, NoAlCorr, **E**: PDT100 TFN-H, **F**: PDT100 TFN-H RepNorm, **G**: TRex-A, **H**: TRex-H, **I**: TRex-H Round-up. The treatments are described in Additional file 1: Table S5.

The time series dataset of 38 activated sludge samples was analyzed in nine different ways to evaluate the effect of different data treatments on the outcome of analyses of the dynamics of the community. The resulting T-RF profiles were then analyzed using Jaccard and Bray-Curtis similarities to assess the stability (Figures 9 and 10) and the rate of change (Additional file 2: Figures S4 and S5) of the bacterial community.

When the stability of the community was assessed by Bray-Curtis similarities there was little difference between most treatments (Figure 9). The treatments that differed were the treatments that did not include correction of the alignment: the *no alignment correction, no normalization* treatment and the three *T-REX* treatments. The *T-REX* treatment that used the simple round-up/down approach to alignment differed the most from the others and suggested that there were much bigger differences (lower Bray-Curtis similarity values) between samples than any of the other treatments. Notably, alignment of T-RF profiles using the round-up/down approach has been used in studies evaluating other aspects of T-RFLP data analysis e.g. ([13,19,27]). As argued by others (e.g. [12]), the results presented here show that the round-up/down approach is not preferable. The other treatments without alignment correction differed the most for a number of samples in the middle of the time series. The difference is due to the erroneous alignment of several T-RFs present in the samples from that period. That the other treatments did not differ very much can be attributed to the abundance distribution of the T-RFs. For all samples the T-RF profile is dominated by a few T-RFs with a relatively high abundance. Since the Bray-Curtis coefficient gives more weight to abundant T-RFs the removal of low abundant T-RFs by the different normalization methods or by applying a higher PDT does not affect the outcome that much. However, when the stability is assessed using Jaccard similarities, which give equal weight to all T-RFs, no treatment results in the same pattern as another (Figure 10).

The rate of change, i.e. how much the community composition changes between each sample, was evaluated the same way as the community stability, using Bray-Curtis (Additional file 2: Figure S4) and Jaccard (Additional file 2: Figure S5) similarities. Likewise, in the assessment using Bray-Curtis similarities (Additional file 2: Figure S4), all treatments except the ones without alignment correction showed similar patterns, and when Jaccard similarities (Additional file 2: Figure S5) were used all treatments resulted in different patterns.

### Ordination analysis

**Figure 11:**
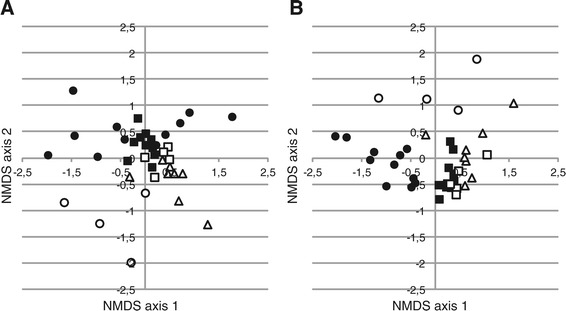
**Impact of data treatment on ordination.** NMDS analysis based on Bray-Curtis similarities of T-RF profiles analyzed with PDT 50 without alignment correction or normalization **(Panel A)** and T-RF profiles analyzed with PDT 100 with alignment correction using the systematic shift procedure and normalization of both replicates and consensus profiles using the TFN-heights procedure **(Panel B)**. The symbols represent the time of sample collection: Filled squares – summer 2003, filled circles – autumn 2003, empty triangles – winter 2004, empty squares – spring/summer 2004, empty circles – late summer 2004.

Different treatments of the T-RF profiles will result in different clustering of samples. Figure 11 shows the NMDS ordination of untreated T-RF profiles and profiles that were treated with alignment correction and normalization of both replicates and consensus profiles. In both ordinations the samples cluster in groups corresponding to the time of sample collection. One group, samples from summer 2003, is spread out and overlaps the other groups in the ordination of untreated data while it clusters more tight and is separated from the other groups in the ordination based on alignment corrected and normalized data. The treatment procedures that include alignment correction all resulted in NMDS ordinations with similar clustering when Bray-Curtis similarities were used. When the ordination was based on Jaccard similarities small differences could be observed between all treatments.

### Correlation analysis with environmental parameters

The correction of ambiguous alignment bins and the removal of T-RFs by normalization change the dynamic pattern of T-RFs in the final alignment bins. Therefore, the type of treatment affects the results of combined analyses of T-RFLP data and environmental parameters. A simple analysis to detect T-RFs that are possibly affected by or might have an effect on environmental parameters is a correlation analysis using T-RF abundance data and environmental parameter data. Table 5 shows the parameters that correlated significantly with four T-RFs after three different treatments. Different conclusions regarding the possible importance of the T-RFs for sludge characteristics would have been drawn from the three different treatments. For example, with treatment A (PDT 50, no alignment correction, no normalization) and C (PDT 100, alignment correction, TFN-heights normalization of both replicates and consensus profiles) the T-RF of size 168 bases showed a statistically significant correlation with the effluent suspended solids, whereas with treatment B (PDT 50, alignment correction, TFN-areas normalization of consensus profiles) it did not. Likewise, the T-RF of size 304 bases showed a statistically significant correlation with the shear sensitivity after treatment A and B but not after treatment C. Which T-RFs, i.e. which groups of bacteria, that would be determined to have a possible role in for example sludge settling properties or floc stability would thus depend on how the data was treated.

**Table 5:** **Correlations between T-RF abundances and WWTP process parameters**

**T-RF size**	**Treatment**	**COD**	**P**	**F/M**	**I**	**H**	**T**	**Fe**	**EtOH**	**PD**	**S**	**SC**	**ESS**
167	A			+									
167	B							+					-
167	C												
168	A	+	+			+						+	+
168	B			+		+	-	-				+	+
168	C	+		+		+	-					+	+
303	A												
303	B				+				+	+			
303	C				+				+	+			
304	A										+		
304	B				-				-		+		
304	C				-				-				

Only correlations significant at the 95% level are shown. + indicate positive correlation and – a negative correlation. Treatment A: PDT 50, No alignment correction, no normalization. Treatment B: PDT 50, Alignment correction using the systematic shift correction procedure, normalization of consensus profiles using the TFN-areas procedure. Treatment C: PDT 100, Alignment correction using the systematic shift correction procedure, normalization of both replicate and consensus profiles using the TFN-heights procedure. In all treatments consensus profiles were generated from two replicate profiles only considering T-RFs present in both. Process parameters: COD - Total COD going into the activated sludge tanks (mg/l), P - Total phosphorus concentration in primary settled wastewater (mg/l), F/M - Food to microorganisms ratio (g/kg*s), I - Inorganic fraction of the activated sludge (%), H - Humic substances in the activated sludge (mg/gMLSS), T - Water temperature, Fe - Total iron dosage, EtOH - Ethanol dosage, PD - Polymer dosage, S - Shear sensitivity of activated sludge flocs, SC - Carbohydrate content in the activated sludge (mg/gMLSS), ESS - Effluent suspended solids (mg/l).

## Conclusions

We acknowledge that some of the conclusions presented here may be specific for the particular system that we used, since the variations in T-RF abundances and sizes are related to the DNA loading and T-RF detection system. However, the discussions regarding alignment and normalization approaches are still relevant, independent of the system used.

We conclude that comparisons of single T-RF profiles from different samples are not reliable, due to variability in detection of low-abundant T-RFs and detection of false T-RFs. Reproducibility can be increased by adapting a high detection threshold and by the combination of two profiles in a consensus profile before comparisons between samples. Normalization of T-RF profiles, both duplicates and consensus profiles, to adjust for the variation in the amount of DNA loaded on the gel, was also shown to contribute to the reproducibility. Of the different normalization methods that were evaluated, all commonly used, the TFN-heights method was the most efficient. Although the arguments for using peak areas as the basis of the analysis of T-RFs are valid, the evaluation presented here show that peak heights are preferred.

There can be a large variation in the estimation of T-RF sizes between samples, which affects the alignment of T-RFs and subsequent comparisons of samples. In alignments of T-RFs from a large number of samples, current automated alignment methods are not entirely reliable, as alignment errors may remain. An additional step, adjusting for systematic shifts in T-RF size estimations between T-RF profiles, was presented here and shown to improve the alignment of T-RFs.

We show that T-RFLP data analysis method choices can determine the conclusions drawn from analyses comparing the community composition between samples, correlation analyses with environmental parameters and analyses of community dynamics patterns. Generally, when T-RFLP is used, at least one of these three analyses is the main tool to answer the proposed research question. This study shows that a conservative approach to normalization and alignment, although resulting in a loss of information, ensures that the results of the T-RFLP analysis are reproducible and reliable.

## Methods

### Sample collection and DNA extraction

Samples were collected at the end of the aerated basins at the Rya WWTP, a WWTP treating both industrial and municipal wastewater [28]. Permission to enter the Rya WWTP and to collect activated sludge samples was granted by Gryaab AB (owner and operator of the WWTP). 50 mL of sample were centrifuged at 4000xg for 3 minutes and the resulting pellet was stored at -20°C within 1.5 h from collection. DNA was extracted using the Power Soil DNA Extraction Kit (MoBio Laboratories). The frozen sludge pellets were thawed, 15 mL sterile water were added and the samples were homogenized by 6 min of mixing in a BagMixer 100 MiniMix (Interscience). Water was removed by centrifugation (4000xg for 3 minutes) and DNA was extracted from 0.25 g of homogenized sludge pellet according to the manufacturer’s instructions.

### PCR for T-RFLP

16S rRNA genes were amplified using HotStarTaqPlus PCR kit (Qiagen) according to the manufacturer’s instructions. The *Bacteria*-specific primer pair 63F (CAGGCCTAACACATGCAAGTC) [29] and M1387R (GGGCGGWGTGTACAAGRC) [30] were used. The forward primer 63F was labeled at the 5’- end with the fluorescent dye 6 – carboxyfluorescein. PCR reactions were carried out in the provided PCR buffer with 0.5 U HotStarTaqPlus, 200 μM dNTP mix, 0.1 μM of each primer and 2-5 ng DNA. The cycle profiles had an initial 5 min at 95°C for Taq polymerase activation followed by 35 cycles of denaturation at 94°C for 1 min, annealing at 60°C for 30 s and elongation at 72°C for 1 min. The reactions were ended with a final elongation step at 72°C for 7 min.

### T-RFLP

The PCR products were purified using the Agencourt AMPure system (Beckman Coulter) and digested with 10 units of restriction enzyme *Hha*I or *Rsa*I (New England Biolabs) in the manufacturer’s provided buffers 4 or 1, respectively. Digestion was carried out at 37°C for at least 16 hours and the restriction digests were purified using the Agencourt AMPure system. For each reaction, 2 μl purified restriction fragments were added to 6.7 μl formamide and 0.3 μl of the size standard LIZ1200 (Applied Biosystems). The fragments were analyzed by capillary gel electrophoresis (3730 DNA Analyzer, Applied Biosystems) using a 20s injection time, a 2.0 kV injection voltage and a 9 kV run voltage. The software GeneMapper (Applied Biosystems) was used to quantify the electropherogram data and to generate the T-RF profiles.

### T-RFLP data analysis

The analysis of the T-RFLP data was carried out using a collection of Visual Basic macros for Microsoft Excel (available at http://sourceforge.net/projects/toolsfortrflp). For comparison, a small number of analyses were also done using the software *T-REX* [16]. Brief descriptions of the different analysis steps are provided here.

### Preparation of data

As a first step in the analysis the analysis range and the peak detection threshold (the lowest acceptable peak height) were defined. In all analyses the peak detection threshold was set to either 50 or 100 fluorescent units and the analysis range was set to 50-1020 bases. The size standard included reference fragments up to 1200 bases. However, proper peaks could not be obtained from the largest fragments and the T-RF sizes could therefore only be estimated up to 1020 bases.

### Normalization of replicate samples

Normalization of replicates was done in five different ways, three of which were based on the total fluorescence normalization (TFN) procedure described by Dunbar *et al.* [12]. The TFN procedure normalizes the profiles so that all profiles get the same total fluorescence, which is defined as either the sum of all peak heights or all peak areas in a profile. The peak heights (or areas) in a profile with a total fluorescence TF which is higher than the lowest total fluorescence in the dataset, TFmin, are re-calculated by multiplication with the factor TFmin/TF. All T-RFs with a peak height (or area) that is below a defined threshold after the multiplication are removed and the total fluorescence, TF, of the profile is re-calculated. The new TF is compared with TFmin again and the process is repeated until TF is equal to TFmin. If the new TF is repeatedly higher than and lower than TFmin, due to the inclusion or exclusion of a T-RF close to the threshold, the profile is calculated taking the average of the two states. In the procedure *TFN-heights*, the total fluorescence is defined as the sum of peak heights and the minimum allowed peak height is the defined peak detection threshold. In the procedure *TFN-areas,* the total fluorescence is defined as the sum of peak areas and the minimum allowed peak area is the minimum observed peak area in the whole dataset. In the procedure *TFN-areas-LT (local threshold),* the total fluorescence is defined as the sum of peak areas and the minimum allowed peak area is the minimum observed peak area among the replicates that are normalized. This procedure is equivalent to the normalization procedure described by Kaplan *et al.* [17].

The two remaining procedures, *FPT-heights* and *FPT-areas*, use an approach where all T-RFs with a relative abundance below a fixed percentage threshold (FPT) are removed. The relative abundance of a T-RF is the peak height (or area) divided by the total fluorescence, i.e. the sum of all peak heights (or areas) in that profile. The FPT approach has previously been described and applied by Li *et al.* using peak areas [22].

### Alignment of replicate samples

#### Automatic alignment

The replicate profiles were aligned using the moving average procedure described by Smith *et al.* [14]. The shortest T-RF of all profiles is identified and placed in an alignment bin. All other T-RFs that are at most Y bases longer than the first T-RF are also included in the alignment bin. The average length of all T-RFs within the alignment bin is then calculated and any additional T-RFs that are at most Z bases longer than the average length of the bin are also included. If a T-RF is added a new average length is calculated and a new search is done to see if more T-RFs are now within the distance Z from the new average and thus should be added. If no additional T-RFs are added a new alignment bin is created and the process starts over with the remaining T-RFs, identifying the shortest T-RF of all profiles that is not already in an alignment bin. A T-RF profile is only allowed to have one T-RF in each alignment bin. In all analyses the parameters Y and Z were set to 1 and 0.5, respectively.

#### Detection and correction of alignment errors

The resulting alignment is not always accurate. If one replicate has a T-RF with a size in between two T-RFs, that both are within the alignment range, in the other replicates, the shortest T-RFs will always be aligned together, even if the longest T-RFs are more similar in size. This happens because the alignment process works from shorter T-RFs to longer T-RFs, without checking if alternative ways of binning the T-RFs are more accurate. For duplicates this is resolved by binning the T-RFs that are most similar in size.

### Generation of consensus profiles

Consensus profiles were generated by combining the data from several replicates. This can be done by calculating the average size, height and areas of the fragments present in either all or a given number of the PCR replicates. In all analyses in this study, consensus profiles were generated from two profiles by only considering T-RFs that were present in both.

### Normalization of consensus profiles

Consensus profiles were normalized using the procedure TFN-areas, TFN-heights, FPT-areas and FPT-heights described above for the normalization of replicate profiles.

### Alignment of consensus profiles

#### Automatic alignment

The consensus profiles were aligned as described for the alignment of replicate profiles. The alignment bins were classified as correct or ambiguous. An alignment bin was classifed as ambiguous if any of the T-RFs in the bin were within the given alignment range of a T-RF in another alignment bin.

#### Detection and correction of systematic differences in size estimation

There are always variations in the size estimation of the T-RFs, even between subsequent loadings of the same sample, and this variation can be the reason why an alignment bin is ambiguous. If the differences in T-RF sizes between two profiles are due to a systematic shift, i.e. if all T-RFs in one of the profiles are shorter than all T-RFs in the other, the T-RF sizes can be recalculated to adjust for the systematic shift and the variation can be reduced. Figure 6 shows a conceptual description of how the procedure works. To correct the T-RF sizes, a T-RF present in all profiles, in an alignment bin classified as correct, is used as a reference and the size of that T-RF is set to the same value in all profiles. All other T-RF sizes are then assigned new values, based on the difference in size compared to the original length of the reference T-RF. The sum of the standard deviations for all unambiguously binned T-RFs are then compared for all possible reference T-RFs, and the reference T-RF that results in the lowest sum of standard deviations is chosen. The profiles are re-aligned as described above and the new alignment can be compared with the original alignment and used as an aid in correcting ambiguous alignment bins.

### Generation of relative abundance data

The relative abundance of a T-RF was calculated as the peak height (or area) divided by the sum of all peak heights (or areas) of the T-RF profile.

### Calculation of association coefficients

Using the relative abundance data two common association coefficients were calculated: the Bray-Curtis distance, which takes the relative abundance of the T-RFs in consideration, and the Jaccard similarity coefficient, which put equal weight on all T-RFs, regardless of their relative abundance (both are described in [31]).

### Datasets

Four datasets were analyzed:

1. Loading duplicates dataset: 51 purified restriction digests were mixed with formamide and size standard. The fragments were analyzed by capillary gel electrophoresis twice.
2. Dilution series dataset: One purified restriction digest was diluted to 17%, 33%, 50%, 67%, 83% and 100% before addition of formamide and size standard.
3. DNA-extraction and PCR replicates dataset: DNA was extracted from an activated sludge sample in two separate reactions. Four PCR reactions were analyzed for each DNA extraction replicate. Two of the PCR replicates for one of the DNA extraction replicates resulted in T-RF profiles with very low total fluorescence and was therefore discarded from the analysis. The four T-RF profiles from the same DNA sample were combined pair-wise in the six possible ways and together with the two T-RF profiles from the other DNA extraction resulted in seven profile pairs.
4. Time series dataset: DNA was extracted from 38 activated sludge samples and 2 PCR replicates were generated and analyzed for each sample.

Table 6 shows a schematic overview of the four datasets.

**Table 6:** **Overview of the generation of the analyzed datasets**

	**Loading duplicates**	**Dilution series**	**DNA-extr. and PCR replicates**	**Time series**
**Sludge samples**				38 samples
**DNA-extraction**			DNA was extracted 2 times from 1 sludge sample	1 DNA-extraction for each sludge sample
**PCR**			4 and 2 PCR reactions from the 2 DNA-extraction replicates	2 PCR reactions for each DNA-extraction
**Restriction digests**		The restriction digest from 1 PCR reaction was diluted to 6 different concentrations.	1 restriction digest per PCR reaction	1 restriction digest per PCR reaction
**Gel loading**	51 restriction digests were loaded 2 times each	6 restriction digests were loaded 2 times each	1 loading per restriction digest	1 loading per restriction digest
**T-RF profiles**	102 profiles	12 profiles	2 + 4 profiles	76 profiles
**Analyzed pairs**	51 pairs	6 pairs	1 + 6 pairs (all possible recombinations of the 4 replicates from the same DNA extraction)	38 pairs

### Dataset analyses

#### Evaluation of differences in size estimation

The loading duplicates dataset was analyzed using a PDT of 50 and aligned as described above. For each duplicate pair the sizes of the T-RFs present in both duplicates were compared.

#### Examples of alignment of T-RFs and systematic shift correction

The time series dataset was analyzed using a PDT of 50. The resulting profiles were analyzed to show how the number of observed T-RFs depends on the total fluorescence of the T-RF profile. PCR replicates were aligned as described above. Consensus profiles were generated only considering T-RFs present in both PCR replicates. The consensus profiles were aligned and systematic shift correction was applied as described above.

#### Evaluation of differences in size estimation

The loading duplicates dataset was analyzed using a PDT of 50 and aligned as described above. For each duplicate pair the sizes of the T-RFs present in both duplicates were compared.

#### Examples of alignment of T-RFs and systematic shift correction

The time series dataset was analyzed using a PDT of 50. The resulting profiles were analyzed to show how the number of observed T-RFs depends on the total fluorescence of the T-RF profile. PCR replicates were aligned and consensus profiles were generated as described above. The consensus profiles were aligned and systematic shift correction was applied as described above.

#### Evaluation of the relation between total fluorescence, DNA concentration and gel loading

The dilution series dataset was analyzed using a PDT of 50 and the total fluorescence was calculated both as the sum of peak heights and peak areas. The loading duplicates dataset was analyzed using a PDT of 50 and the total fluorescence was calculated as the sum of peak heights.

#### Examples of how differences in total fluorescence affects the comparisons of T-RF profiles

The dilution series dataset was analyzed using a PDT of 50. All profiles were aligned together as described above. Relative abundances of T-RFs and Bray-Curtis similarities were calculated based on peak heights. Jaccard similarities were calculated. The loading duplicates dataset was analyzed using either a PDT of 50 or 100. The duplicate profiles were aligned as described above. Relative abundances of T-RFs and Jaccard and Bray-Curtis similarities were calculated based on either peak heights or peak areas.

#### Evaluation of normalization procedures

The loading duplicates dataset was analyzed using either a PDT of 50 or 100. The duplicate profiles were normalized using the five different procedures described above: TFN-heights, TFN-areas, TFN-areas-LT, FPT-heights and FPT-areas. After normalization, the duplicate profiles were aligned as described above. Relative abundances of T-RFs and Jaccard and Bray-Curtis similarities were calculated based on either peak heights or peak areas, depending on which of the two the normalization procedure was based on. The statistical significance of the differences between the treatments was calculated using a Kruskal-Wallis test followed by Mann-Whitney pairwise comparisons with Bonferroni corrected P-values. For both tests a significance level of 0.05 was used.

#### Evaluation of the effect of using single profiles or consensus profiles

The dilution series dataset was analyzed using a PDT of either 50 or 100. The first (Run 1) and second (Run 2) gel loadings were either analyzed separately or together, using the loading duplicates to generate consensus profiles. Alignment of the T-RF profiles was carried out as described above. The total fluorescence was calculated as the sum of peak heights and the TFN-heights procedure was used for normalization of both single profiles, loading duplicate profiles, and consensus profiles.

#### Evaluation of combinations of PDT, alignment correction and normalization

The DNA-extraction and PCR replicates dataset was analyzed in 18 different ways (described in Additional file 1: Table S5). Four of the analyses used the *T-REX* software [16], with the following settings: Noise filtering was performed using the procedure described by Abdo *et al.* [15] for all samples based on either peak heights or peak areas. The standard deviation multiplier was set to 1. The T-RFs were aligned either by using the T-align method [14] with a clustering threshold of 1, allowing at most one peak per plot in each T-RF, or by rounding every peak size up or down to the nearest integer. After alignment a data matrix was constructed using the average peak height or area data of the replicates. The relative abundance of each T-RF was then calculated as the peak height (or area) of the T-RF divided by the sum of all peak heights (or areas) in the T-RF profile.

#### Evaluation of normalization efficiency

The dilution series was analyzed using a PDT of 100, and using the two loading duplicates for each dilution to create consensus profiles. Both loading duplicates and consensus profiles were aligned as described above. Both loading duplicates and consensus profiles were normalized using the TFN-heights procedure. Relative abundances of T-RFs and Jaccard and Bray-Curtis similarities were calculated based on peak heights. The DNA-extraction and PCR replicate dataset was analyzed using a PDT of 100. Both replicates and consensus profiles were normalized using the TFN-heights procedure. Both replicates and consensus profiles were aligned as described above. The alignment of the consensus profiles was further corrected using the systematic shift correction procedure. Relative abundances of T-RFs and Jaccard and Bray-Curtis similarities were calculated based on peak heights. The loading duplicate dataset was analyzed using a PDT of 50. The duplicates were normalized using the TFN-heights procedure. Relative abundances of T-RFs and Jaccard and Bray-Curtis similarities were calculated based on peak heights.

#### Evaluation of the impact of different treatment methods on bacterial dynamics

The time series dataset was analyzed in nine different ways: PDT50 TFN-A, PDT50 TFN-H, PDT50 NoNorm, PDT50 NoNorm NoAlCorr, PDT100 TFN-H, PDT100 TFN-H RepNorm, TRex-A, TRex-H and TRex-H Round-up. The treatments are described in Additional file 1: Table S5. Bray-Curtis and Jaccard similarities between all T-RF profiles were calculated.

#### Evaluation of the impact of different treatment methods on the outcome of an ordination analysis

The time series dataset, analyzed as in the evaluation of the impact of different treatment methods on bacterial dynamics, was used. Non-metric multi-dimensional scaling analysis (MDS) of Bray-Curtis and Jaccard distance matrices was carried out using the software Primer 6 (Primer-E). The Jaccard distance was calculated as (1 – Jaccard similarity). The analysis was performed using 100 repetitions, Kruskal stress formula number 1 and a minimum stress of 0.01.

#### Evaluation of the impact of different treatment methods on the outcome of a correlation analysis

The time series dataset, analyzed as in the evaluation of the impact of different treatment methods on bacterial dynamics, was used. The Pearson’s product momentum correlation coefficient was used to estimate the linear correlation between relative abundances of T-RFs, process parameters and sludge properties. To determine the statistical significance of the correlation a t-test was carried out using a significance level of 0.05.

### Availability of supporting data

All supporting data, T-RF profiles for the four datasets used in the analyses, are provided in Additional file 3.

## Additional files

Additional file 1: Table S1. Examples of automatic and corrected alignment of T-RFs in two samples. **Table S2.** An example of automatic and corrected alignment of T-RFs in four samples. **Table S3.** Number of T-RFs and Jaccard and Bray-Curtis similarities for single and consensus profiles in the dilution series dataset. **Table S4.** Number of T-RFs and Jaccard and Bray-Curtis similarities of the DNA-extraction and PCR replicates dataset after different treatments. **Table S5.** Overview of the analysis combinations applied to the DNA-extraction and PCR replicates dataset. Additional file 2: Figure S1. Number of T-RFs versus total fluorescence. **Figure S2.** Example of removal of false peaks by increasing the PDT. **Figure S3.** Similarity versus difference in total fluorescence. **Figure S4.** Rate of change: Bray-Curtis similarity between subsequent T-RF profiles. **Figure S5.** Rate of change: Jaccard similarity between subsequent T-RF profiles. Additional file 3:
**Supporting data.**
