# Supplementary material for: Impact of T-RFLP data analysis choices on assessments of microbial community structure and dynamics
Source: BMC Bioinformatics. 2014 Nov 8;15(1):360. doi: 10.1186/s12859-014-0360-8 (PMC4232699; doi:10.1186/s12859-014-0360-8)
Supplement: Additional file 1: Table S1. — Examples of automatic and corrected alignment of T-RFs in two samples. Table S2. An example of automatic and corrected alignment of T-RFs in four samples. Table S3. Number of T-RFs and Jaccard and Bray-Curtis similarities for single and consensus profiles in the dilution series dataset. Table S4. Number of T-RFs and Jaccard and Bray-Curtis similarities of the DNA-extraction and PCR replicates dataset after different treatments. Table S5. Overview of the analysis combinations applied to the DNA-extraction and PCR replicates dataset. [file 12859_2014_360_MOESM1_ESM.doc]

**Table S1 Examples of automatic and corrected alignment of T-RFs in two samples**

|  | **Automatic alignment** | | **Corrected alignment** | |
| --- | --- | --- | --- | --- |
|  | **Sample1** | **Sample2** | **Sample1** | **Sample2** |
| **T-RF 1** | 303.53 | 304.33 | 303.53 | 0 |
| **T-RF 2** | 304.41 | 0 | 304.41 | 304.33 |
| **T-RF 3** | 473.29 | 472.41 | 0 | 472.41 |
| **T-RF 4** | 0 | 473.34 | 473.29 | 473.34 |

The given numbers are the T-RF sizes in bases**.**

Table S2 An example of automatic and corrected alignment of T-RFs in four samples

|  | **Automatic alignment** | | | | **Corrected alignment** | | | |
| --- | --- | --- | --- | --- | --- | --- | --- | --- |
|  | **Sample1** | **Sample2** | **Sample3** | **Sample4** | **Sample1** | **Sample2** | **Sample3** | **Sample4** |
| **T-RF 1** | 164.28 | 163.92 | 0 | 164.77 | 164.28 | 163.92 | 0 | 0 |
| **T-RF 2** | 0 | 0 | 164.98 | 0 | 0 | 0 | 164.98 | 164.77 |

The given numbers are the T-RF sizes in bases**.**

**Table S3 Number of T-RFs and Jaccard and Bray-Curtis similarities for single and consensus profiles in the dilution series data**set

|  | **Number of T-RFs** | | | | **Jaccard** | | | | **Bray-Curtis** | | | |
| --- | --- | --- | --- | --- | --- | --- | --- | --- | --- | --- | --- | --- |
| **Treatment** | **Average** | **StDev** | **Min** | **Max** | **Average** | **StDev** | **Min** | **Max** | **Average** | **StDev** | **Min** | **Max** |
| **PDT50 TFN-H Single Run 1** | 12.8 | 5.5 | 9 | 22 | 71.0% | 24.4% | 33.3% | 90.0% | 88.4% | 12.8% | 66.7% | 97.1% |
| **PDT50 TFN-H Single Run 2** | 14.6 | 5.5 | 11 | 24 | 73.4% | 28.2% | 29.6% | 100.0% | 87.4% | 14.1% | 62.8% | 97.0% |
| **PDT50 TFN-H RepNorm Consensus** | 11.2 | 3.8 | 9 | 18 | 79.5% | 27.3% | 35.0% | 100.0% | 91.2% | 11.8% | 70.4% | 98.7% |
| **PDT100 TFN-H Single Run 1** | 4.4 | 0.5 | 4 | 5 | 92.0% | 11.0% | 80.0% | 100.0% | 96.2% | 3.2% | 92.5% | 99.1% |
| **PDT100 TFN-H Single Run 2** | 4.2 | 0.4 | 4 | 5 | 96.0% | 8.9% | 80.0% | 100.0% | 96.9% | 4.0% | 89.8% | 99.1% |
| **PDT100 TFN-H RepNorm Consensus** | 4.2 | 0.4 | 4 | 5 | 96.0% | 8.9% | 80.0% | 100.0% | 97.6% | 2.8% | 92.6% | 99.3% |

Run 1 and run 2 were two separate loadings of the same sample. Treatments: PDT (Peak detection threshold), TFN-H (Total fluorescence normalization with total fluorescence defined as sum of all peak heights), RepNorm (Normalization of replicates before alignment and generation of consensus profiles using the same normalization procedure as given for the consensus profiles), Single (Analysis of single T-RF profiles, no generation of consensus profiles). Consensus (Consensus profiles generated from the profiles of run 1 and 2 only considering T-RFs present in both). Both replicate profiles and consensus profiles were aligned using the moving average procedure. The Jaccard and Bray-Curtis similarities are the similarities with the profile of the undiluted sample.

**Table S4 Number of T-RFs and Jaccard and Bray-Curtis similarities of the DNA-**extraction and PCR replicates data set after different treatments

|  | **Number of T-RFs** | | | | **Jaccard** | | | | **Bray-Curtis** | | | |
| --- | --- | --- | --- | --- | --- | --- | --- | --- | --- | --- | --- | --- |
| **Treatment** | **Average** | **StDev** | **Min** | **Max** | **Average** | **StDev** | **Min** | **Max** | **Average** | **StDev** | **Min** | **Max** |
| **PDT50 NoAlCorr NoNorm** | 29.0 | 1.8 | 27.0 | 32.0 | 86.3% | 7.5% | 73.5% | 93.8% | 94.5% | 3.9% | 87.0% | 97.8% |
| **PDT50 NoNorm** | 28.1 | 1.7 | 26.0 | 31.0 | 88.3% | 4.3% | 83.9% | 93.5% | 95.7% | 2.7% | 90.4% | 97.8% |
| **PDT50 TFN-H** | 26.3 | 1.4 | 24.0 | 28.0 | 90.6% | 3.6% | 85.7% | 96.4% | 96.0% | 2.8% | 90.5% | 97.8% |
| **PDT50 TFN-H RepNorm** | 24.9 | 1.1 | 23.0 | 26.0 | 87.7% | 3.8% | 81.5% | 92.6% | 95.5% | 2.5% | 90.6% | 97.6% |
| **PDT50 TFN-A** | 26.0 | 1.8 | 23.0 | 28.0 | 88.8% | 6.8% | 78.6% | 96.4% | 95.9% | 3.0% | 89.9% | 98.0% |
| **PDT50 FPT-H** | 21.0 | 1.9 | 17.0 | 23.0 | 87.2% | 8.4% | 73.9% | 95.7% | 95.5% | 3.4% | 89.0% | 97.8% |
| **PDT50 FPT-A** | 18.0 | 1.6 | 15.0 | 20.0 | 85.6% | 7.9% | 76.2% | 94.4% | 95.2% | 2.7% | 90.6% | 97.8% |
| **PDT50 Single TFN-H** | 26.0 | 2.0 | 23.0 | 29.0 | 84.4% | 3.2% | 81.5% | 89.3% | 92.3% | 2.0% | 89.9% | 94.7% |
| **PDT50 TRex-A** | 34.6 | 5.5 | 28.0 | 41.0 | 79.8% | 15.1% | 65.9% | 100.0% | 92.5% | 6.6% | 79.2% | 96.7% |
| **PDT50 TRex-H** | 32.0 | 4.9 | 26.0 | 37.0 | 82.7% | 13.6% | 70.3% | 100.0% | 92.7% | 7.2% | 78.2% | 96.7% |
| **PDT50 TRex-H Round-up** | 36.0 | 5.7 | 28.0 | 43.0 | 70.8% | 18.3% | 44.4% | 90.9% | 80.3% | 17.3% | 46.3% | 93.0% |
| **PDT50 Abdo** | 23.6 | 1.3 | 22.0 | 26.0 | 84.4% | 4.2% | 77.8% | 88.5% | 95.2% | 2.8% | 89.6% | 97.0% |
| **PDT100 NoAlCorr NoNorm** | 15.7 | 2.9 | 14.0 | 22.0 | 65.5% | 6.3% | 56.5% | 72.7% | 89.4% | 2.6% | 84.6% | 91.8% |
| **PDT100 NoNorm** | 15.7 | 2.9 | 14.0 | 22.0 | 66.7% | 4.7% | 63.6% | 72.7% | 90.6% | 2.1% | 87.1% | 92.7% |
| **PDT100 TFN-H** | 14.0 | 0.6 | 13.0 | 15.0 | 92.2% | 2.7% | 86.7% | 93.3% | 96.4% | 2.8% | 90.8% | 98.0% |
| **PDT100 TFN-H RepNorm** | 13.3 | 0.8 | 12.0 | 14.0 | 95.1% | 3.8% | 92.3% | 100.0% | 96.6% | 2.4% | 92.0% | 99.0% |
| **PDT100 TFN-A** | 14.9 | 1.5 | 14.0 | 18.0 | 71.7% | 3.9% | 68.4% | 77.8% | 92.8% | 2.0% | 89.2% | 94.7% |
| **PDT100 FPT-H** | 15.7 | 2.9 | 14.0 | 22.0 | 66.7% | 4.7% | 63.6% | 72.7% | 90.6% | 2.1% | 87.1% | 92.7% |
| **PDT100 FPT-A** | 14.9 | 1.5 | 14.0 | 18.0 | 71.7% | 3.9% | 68.4% | 77.8% | 92.8% | 2.0% | 89.2% | 94.7% |
| **PDT100 Single TFN-H** | 13.5 | 1.4 | 11.0 | 15.0 | 82.6% | 3.8% | 78.6% | 86.7% | 92.8% | 2.3% | 90.1% | 94.8% |

The treatments are described in Table S5. The Jaccard and Bray-Curtis similarities are the similarities with the profile with the highest total fluorescence.

**Table S5 Overview of the analysis combinations applied to the DNA-extraction and PCR replicates dataset**

|  | PDT | Analysis based on peak heights (H) or areas (A) | Noise removal^a^ | Analysis based on single (S) or consensus (C) profiles | Normalization of replicates^b^ | Alignment of replicates^c^ | Alignment correction^d^ | Normalization of consensus profiles^e^ | Alignment of consensus profiles^c^ | Alignment correction^f^ |
| --- | --- | --- | --- | --- | --- | --- | --- | --- | --- | --- |
| **PDT50 NoAlCorr NoNorm** | 50 | H | No | C | No | MovAvg | No | No | MovAvg | No |
| **PDT50 NoNorm** | 50 | H | No | C | No | MovAvg | Yes | No | MovAvg | Yes |
| **PDT50 TFN-H** | 50 | H | No | C | No | MovAvg | Yes | TFN | MovAvg | Yes |
| **PDT50 TFN-H RepNorm** | 50 | H | No | C | TFN | MovAvg | Yes | TFN | MovAvg | Yes |
| **PDT50 TFN-A** | 50 | A | No | C | No | MovAvg | Yes | TFN | MovAvg | Yes |
| **PDT50 FPT-H** | 50 | H | No | C | No | MovAvg | Yes | FPT | MovAvg | Yes |
| **PDT50 FPT-A** | 50 | A | No | C | No | MovAvg | Yes | FPT | MovAvg | Yes |
| **PDT50 Single TFN-H** | 50 | H | No | S |  |  |  | TFN | MovAvg | Yes |
| **PDT50 TRex-A** | 50 | A | Yes | C | No | MovAvg | No | No | MovAvg | No |
| **PDT50 TRex-H** | 50 | H | Yes | C | No | MovAvg | No | No | MovAvg | No |
| **PDT50 TRex-H Round-up** | 50 | H | Yes | C | No | Round-up | No | No | Round-up | No |
| **PDT50 Abdo** | 50 | H | Yes | C | No | MovAvg | No | No | MovAvg | No |
| **PDT100 NoAlCorr NoNorm** | 100 | H | No | C | No | MovAvg | No | No | MovAvg | No |
| **PDT100 NoNorm** | 100 | H | No | C | No | MovAvg | Yes | No | MovAvg | Yes |
| **PDT100 TFN-H** | 100 | H | No | C | No | MovAvg | Yes | TFN | MovAvg | Yes |
| **PDT100 TFN-H RepNorm** | 100 | H | No | C | TFN | MovAvg | Yes | TFN | MovAvg | Yes |
| **PDT100 TFN-A** | 100 | A | No | C | No | MovAvg | Yes | TFN | MovAvg | Yes |
| **PDT100 FPT-H** | 100 | H | No | C | No | MovAvg | Yes | FPT | MovAvg | Yes |
| **PDT100 FPT-A** | 100 | A | No | C | No | MovAvg | Yes | FPT | MovAvg | Yes |
| **PDT100 Single TFN-H** | 100 | H | No | S |  |  |  | TFN | MovAvg | Yes |

a) Noise removal using the method by Abdo et al. [1] which is included in the T-REX software [2]. b) TFN – Total fluorescence normalization procedure. c) MovAvg – The moving average procedure, Round-up – The round up/down approach included in the T-REX software. d) Correction of alignment of replicate profiles as described in this study. e) TFN – Total fluorescence normalization procedure, FPT – Fixed percentage threshold procedure. f) Alignment correction using the systematic shift correction procedure described in this study.

**References**

1. Abdo Z, Schüette UM, Bent SJ, Williams CJ, Forney LJ, Joyce P: **Statistical methods for characterizing diversity of microbial communities by analysis of terminal restriction fragment length polymorphisms of 16S rRNA genes**. *Environmental Microbiology* 2006, **8**(5):929-938.

2. Culman S, Bukowski R, Gauch H, Cadillo-Quiroz H, Buckley D: **T-REX: software for the processing and analysis of T-RFLP data**. *BMC Bioinformatics* 2009, **10**(1):171.
