# Supplementary material for: Impact of T-RFLP data analysis choices on assessments of microbial community structure and dynamics
Source: BMC Bioinformatics. 2014 Nov 8;15(1):360. doi: 10.1186/s12859-014-0360-8 (PMC4232699; doi:10.1186/s12859-014-0360-8)
Supplement: Additional file 2: Figure S1. — Number of T-RFs versus total fluorescence. Figure S2. Example of removal of false peaks by increasing the PDT. Figure S3. Similarity versus difference in total fluorescence. Figure S4. Rate of change: Bray-Curtis similarity between subsequent T-RF profiles. Figure S5. Rate of change: Jaccard similarity between subsequent T-RF profiles. [file 12859_2014_360_MOESM2_ESM.doc]

**Figure S1 Number of T-RFs versus total fluorescence**Number of T-RFs after treatment plotted against total fluorescence (sum of peak heights) before treatment of 102 T-RF profiles from the loading duplicates dataset. The T-RF profiles were analyzed using a peak detection threshold (PDT) of 50. The treatments are described in the methods section in the main manuscript.


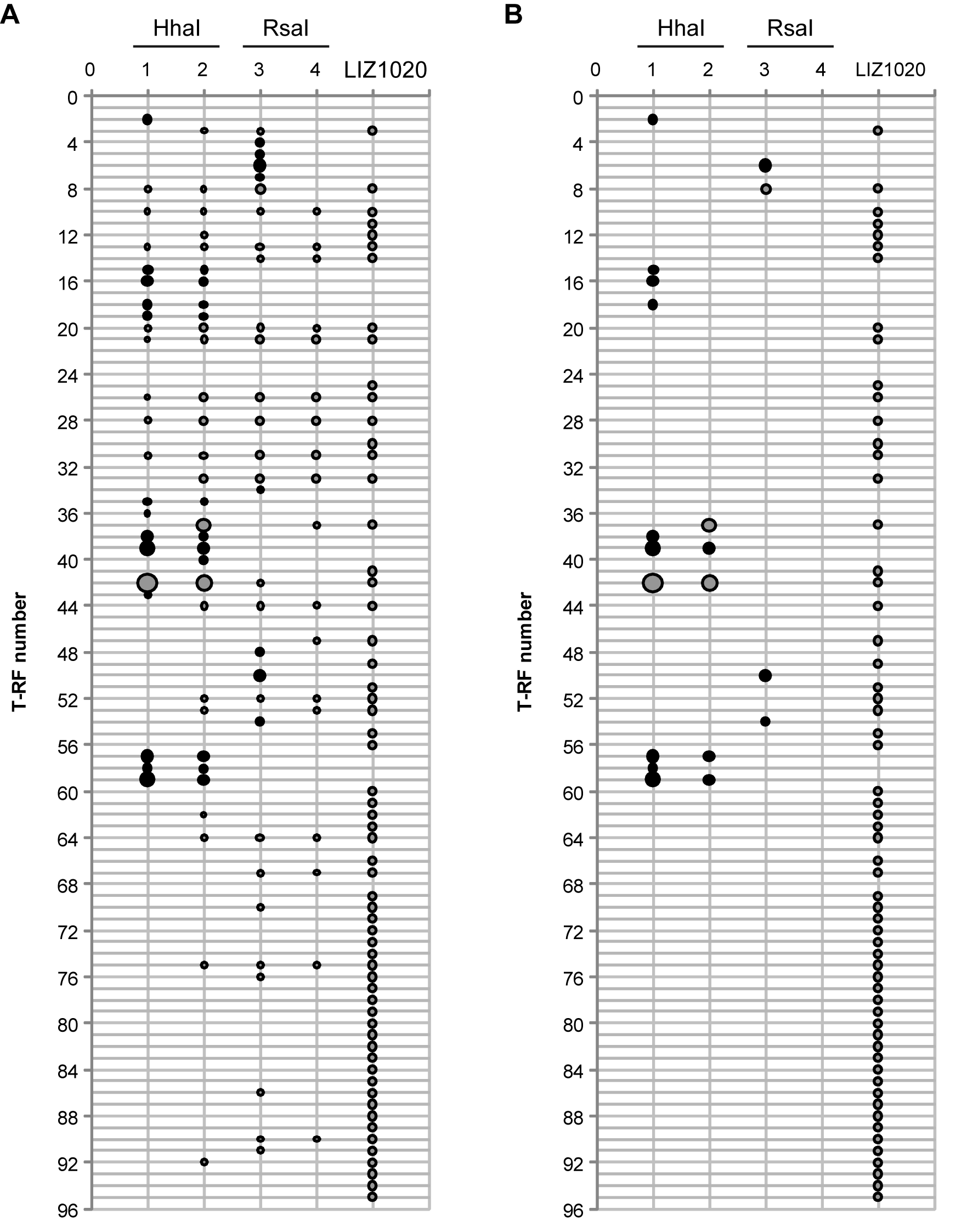


**Figure S2 Example of removal of false peaks by increasing the PDT**T-RFLP analysis of a single sample with two restriction enzymes and two PCR duplicates for each enzymatic reaction. The analysis was carried out using a PDT of 50 (panel A), which included false T-RFs produced by spectral pull-up, and a PDT of 100 (panel B), which removed the false T-RFs. The size of the circles represent the relative abundance (based on peak heights) of the T-RFs. Gray circles are T-RFs of the same size as the size standard.

**Figure S3 Similarity versus difference in total fluorescence**
Analysis of the loading duplicate dataset. A PDT of 100 was applied and the duplicates were normalized using the TFN-heights procedure. Columns – total fluorescence (sum of peak heights) of the duplicate with the lowest total fluorescence before treatment as percentage of the total fluorescence of the duplicate with the highest total fluorescence. Circles – Jaccard similarity of the two duplicates. Squares – Bray-Curtis similarity of the two duplicates.


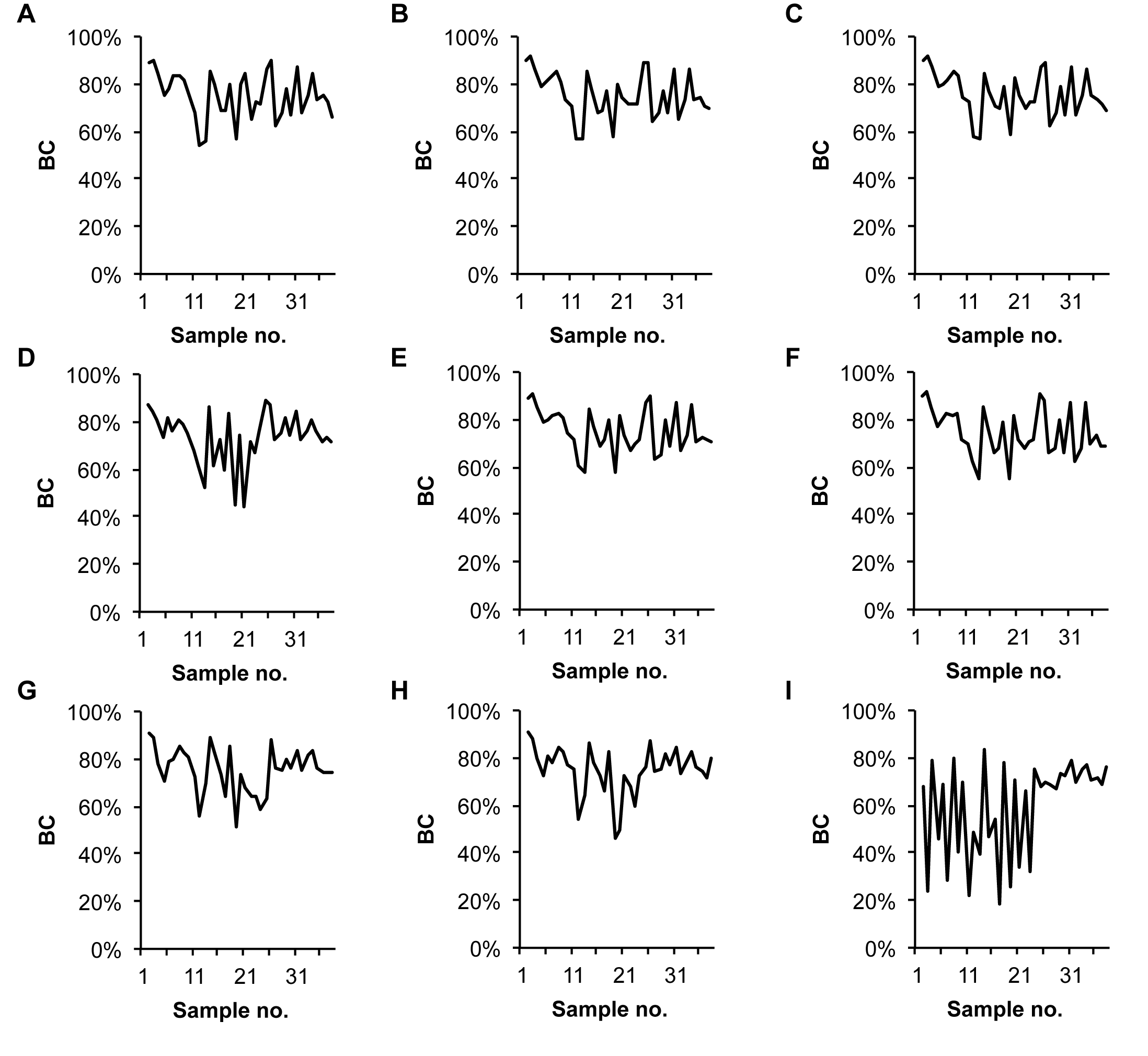


**Figure S4 Rate of change: Bray-Curtis similarity between subsequent T-RF profiles**The Bray-Curtis similarity (BC) between each sample and the preceding sample is plotted for all samples. The data was treated in nine different ways before calculation of Bray-Curtis similarities. Panel A: PDT50 TFN-A, B: PDT50 TFN-H, C: PDT 50 NoNorm, D: PDT50 NoNorm, NoAlCorr, E: PDT100 TFN-H, F: PDT100 TFN-H RepNorm, G: TRex-A, H: TRex-H, I: TRex-H Round-up. The treatments are described in Table S5.


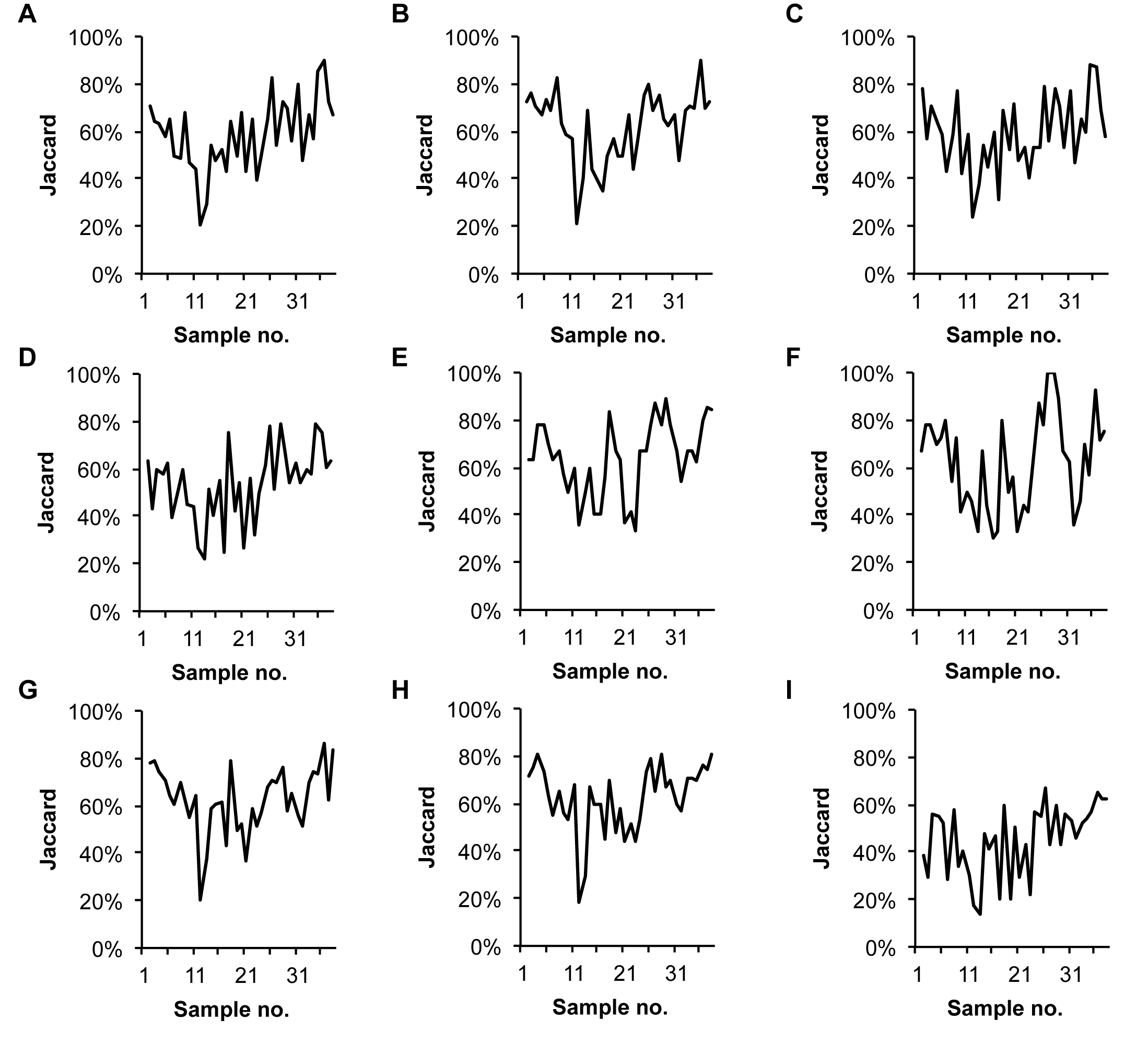


**Figure S5 Rate of change: Jaccard similarity between subsequent T-RF profiles**The Jaccard similarity (Jaccard) between each sample and the preceding sample is plotted for all samples. The data was treated in nine different ways before calculation of Jaccard similarities. Panel A: PDT50 TFN-A, B: PDT50 TFN-H, C: PDT 50 NoNorm, D: PDT50 NoNorm, NoAlCorr, E: PDT100 TFN-H, F: PDT100 TFN-H RepNorm, G: TRex-A, H: TRex-H, I: TRex-H Round-up. The treatments are described in Table S5.
